# Comment on “Prospective multicenter validation of a machine learning model for predicting anastomotic leakage in patients with gastric adenocarcinoma undergoing total or proximal gastrectomy”

**DOI:** 10.1097/JS9.0000000000004794

**Published:** 2026-01-13

**Authors:** Jingjing Zhong

**Affiliations:** Reproductive Medicine Center, Hainan Women and Children’s Medical Center, Haikou, China

*To the Editor,*

Shao *et al* report a prospective multicenter validation of a real-time, web-based machine learning (ML) model to predict anastomotic leakage (AL) after total or proximal gastrectomy for gastric adenocarcinoma[1]. In four Chinese centers, 512 consecutive patients were enrolled; AL occurred in 13 (2.54%). The model, previously trained on 1660 retrospective cases, achieved an area under the receiver operating characteristic curve of 0.78 in the validation cohort, with sensitivity of 0.769, specificity of 0.577, and a negative predictive value of 0.990. Of note, a risk threshold of 0.45 identified roughly one-third of patients who experienced no AL and could potentially forgo intensive monitoring[1]. In preparing this commentary, we followed the TITAN 2025 recommendations for transparent and responsible reporting of surgical research that may involve AI-based tools[2].

This work addresses a clinically important problem. AL after total or proximal gastrectomy remains one of the most feared complications in upper gastrointestinal surgery, associated with sepsis, re-intervention, prolonged hospital stay, and worse oncologic outcomes[3]. Traditional scores such as Tu’s nomogram incorporate demographic, nutritional, and operative variables but show only moderate discrimination and are not designed for real-time intraoperative use[4]. Shao *et al* move beyond retrospective modeling by prospectively embedding their ML tool (https://gasal.21cloudbox.com/) into the operative workflow and testing it across multiple centers, an important step toward real-world implementation.

The study has several strengths. Consecutive enrollment, predefined inclusion criteria, and STROCSS-concordant reporting enhance internal validity. The model uses routinely available clinical variables (e.g. albumin, hemoglobin, tumor size, ASA class, and operative time), which supports scalability. Performance is reported not only overall but also separately for the derivation-like “TJ” cohort and external centers, with AUCs of 0.760 and 0.940, respectively[1]. Importantly, the post hoc threshold analysis translates statistical performance into a tangible clinical proposition: safely de-escalating postoperative surveillance for approximately 35% of low-risk patients.

However, several issues temper immediate adoption. First, only 13 AL events were observed. This extreme class imbalance inevitably limits the stability of performance estimates and the reliability of feature importance rankings. The modest positive predictive value (≈4%–6%) means most “high-risk” alerts will be false positives, raising the risk of alarm fatigue and unnecessary investigations. Second, all centers were from one country and one health care system; model performance in non-Asian populations, in lower-volume hospitals, or with different reconstruction techniques remains unknown. Third, the study does not compare the ML tool against surgeons’ gestalt or existing scores in a head-to-head fashion, nor does it show that using the model changes management or outcomes – key criteria for clinical utility[5].

For now, this model is best viewed as a promising decision-support tool rather than a definitive gatekeeper for monitoring intensity. Future research should focus on multinational external validation, calibration, and decision-curve analysis; prospective impact studies testing protocolized responses to model outputs; and integration into electronic health record systems with clear governance for AI-assisted decision-making.

Shao *et al* should be congratulated for moving AL prediction from static retrospective models toward prospective, workflow-embedded AI. Their study provides an important foundation; the next step is to demonstrate that ML-guided risk stratification can safely reallocate resources and improve outcomes for patients undergoing major gastric surgery.

## Data Availability

No data were used in this Letter to the Editor.
